# Establishing a Chemical Component Relaxation Service Using Botulinum Toxin For Abdominal Wall Reconstruction: Single-Centre Experience From a UK Tertiary Hernia Referral Unit

**DOI:** 10.3389/jaws.2025.14976

**Published:** 2025-11-17

**Authors:** Lawrence Nip, Oliver Curwen, Sarah Zhao, Ravi Jakkalasaibaba, Steve Halligan, Alastair C. J. Windsor, Samuel G. Parker, Rhys Thomas

**Affiliations:** 1 The Abdominal Wall Unit, Croydon University Hospital, London, United Kingdom; 2 Centre for Medical Imaging, Division of Medicine, University College London, The Rayne Institute, London, United Kingdom; 3 The Princess Grace Hospital, HCA Healthcare, London, United Kingdom

**Keywords:** downstaging, abdominal wall reconstruction, component separation, complex hernia, botulinum toxin A

## Abstract

**Background:**

Botulinum toxin type A (BTA) is a valuable adjunct in abdominal wall reconstruction (AWR). Chemical component relaxation (CCR) involves injecting BTA into the lateral abdominal wall, leading to muscle paralysis and elongation which facilitates primary fascial closure during surgery without the need for extensive dissection. There are currently no standardised protocols for BTA administration in the perioperative period for AWR. We present a standardised protocol for CCR from our tertiary hernia unit and report our outcomes following surgery.

**Methods:**

A retrospective analysis of a prospective dataset of all patients undergoing standardised pre-operative CCR between 1^st^ May 2021 and 30^th^ April 2024 for AWR were included in this study. Analysis of pre-operative multi-disciplinary team (MDT) planning, BTA administration, surgical procedure and outcomes were performed.

**Results:**

During the 3-year-period, 35 patients underwent CCR with subsequent AWR. The median age was 58 and median BMI was 32. Median hernia defect width was 8 cm. Anterior and posterior sheath closure was achieved in 91% of cases. In total, 39% with defect size >8 cm did not require component separation and were considered “downstaged”. There were no complications following CCR, and the surgical site occurrence rate following AWR was 26%. Hernia recurrence occurred in 1 patient.

**Conclusion:**

The presented protocol of pre-operative BTA appears to be a safe method of CCR. We demonstrate that its use may reduce the need for component separation and is associated with good post-operative outcomes.

## Introduction

Incisional hernias are increasingly common with occurrence rates of 9%–20% after primary laparotomy [1]. Within the surgeon’s armamentarium for repair of large incisional hernias are the anterior and posterior component separation techniques described by Ramirez [2] and Novitsky [3]. These procedures, particularly transversus abdominis release (TAR), are challenging to perform, have steep learning curves, and carry elevated risk of complications. Moreover, muscle-releasing incisions interfere with abdominal wall integrity and core stability as the divided muscles atrophy over time.

Botulinum Toxin A (BTA) is now an established adjunct for abdominal wall reconstruction (AWR) as a chemical component relaxation (CCR) technique. It is a neurotoxin derived from *Clostridium Botulinum* that selectively blocks the release of acetylcholine from presynaptic cholinergic terminals to prevent nerve conduction. Its use was first presented in 2009 by the Mexican group Ibarra-Hurtado and colleagues [4]. Their group described 5 injections bilaterally in the lateral abdominal wall of 12 patients and demonstrated a reduction in mean hernia defect width of 5.25 cm after 4 weeks. Notably, half did not require intraoperative component separation.

Over the subsequent 15 years, the use of BTA has grown in popularity, with literature supporting its use in the pre-operative setting prior to AWR worldwide [5]. Administration of BTA can help achieve primary fascial closure (PFC) of both anterior and posterior rectus sheaths by flaccid paralysis and elongation of the lateral muscle complex, which facilitates medialisation during surgery. Some authors suggest that it can be used as the primary modality to assist with PFC [6, 7] rather than classic approaches of anterior or posterior component separation. It also confers an advantage of being non-invasive and can be performed in the outpatient setting. A systematic review of 995 patients demonstrated that BTA allowed an average elongation of lateral abdominal wall muscles by 3.2 cm bilaterally and was associated with a significant improvement in fascial closure rates [8].

However, the heterogeneity in techniques and regimens used for preoperative BTA is a notable concern. Uncertainty remains regarding its dosing, formulation, timing, number of injections, injection sites, and adjunctive techniques. Published data remains limited, with few protocols reported. Some studies fail to rigorously report on key technical steps such as number of injections, anatomical site, specific indication for the use of BTA and role of clinical governance to ensure patient safety. Despite rapidly evolving interest, there is no current standardised protocol for the administration of BTA. In this article, we therefore share our experience of establishing a BTA service in a UK tertiary hernia unit, with the aim of proposing a safe, reproducible, and standardised administration protocol.

## Materials and Methods

### Type of Study and Participants

This was a retrospective single-centre case series. Consecutive adult patients aged 18 years or older who underwent (BTA) administration for chemical component relaxation (CCR) were prospectively collected for inclusion in the database, which was retrospectively maintained and analysed for outcomes. The period of data collection was 1st May 2021 to 30th April 2024, which provided a 3-year overview of the development of a standardised service. Patients with known contraindications to BTA such as myasthenia gravis, multiple sclerosis, severe COPD, breastfeeding, and pregnancy were excluded from our study. For the analysis, patients undergoing parastomal hernia repair, flank hernia repair and those that were referred just for BTA injection (having subsequent AWR elsewhere) were excluded. Included patients therefore had ventral hernias and received both BTA and AWR at our centre.

### Establishing a Chemical Component Relaxation Service

The experience of establishing a protocol for BTA is described by authors SP and RT, as the senior authors and joint leads of our tertiary hernia centre. When building the CCR service, there were three main areas of focus: 1) hospital board approval; 2) maintaining a safe patient pathway; and 3) presenting all outcomes for review in clinical governance meetings.

#### Board Approval

In the UK, BTA is not licensed for CCR, therefore, approval from the local hospital board and medicines approval committee was necessary to allow regulated off-label dispensing and clinical use. Presentation of the literature supporting the use of BTA was presented to the committees, with approval from the hospital board, lead pharmacist and the elective surgery leads. In response to the committee’s challenge to source an algorithm to determine cases where BTA is appropriate, the senior author (RT) discussed with consultants from 5 other AWR tertiary referral units in the UK. Anecdotally surgeons were unaware of such algorithms being in use, and it was down to the local multi-disciplinary team (MDT) to decide. However, there was consensus that hernia defect widths exceeding 10 cm and loss of domain more than 25% of the intraabdominal volume (Sabbagh method [9]) were indications for BTA. This was supported by a protocol published by Yurtkap et al. [10].

**FIGURE 1:**
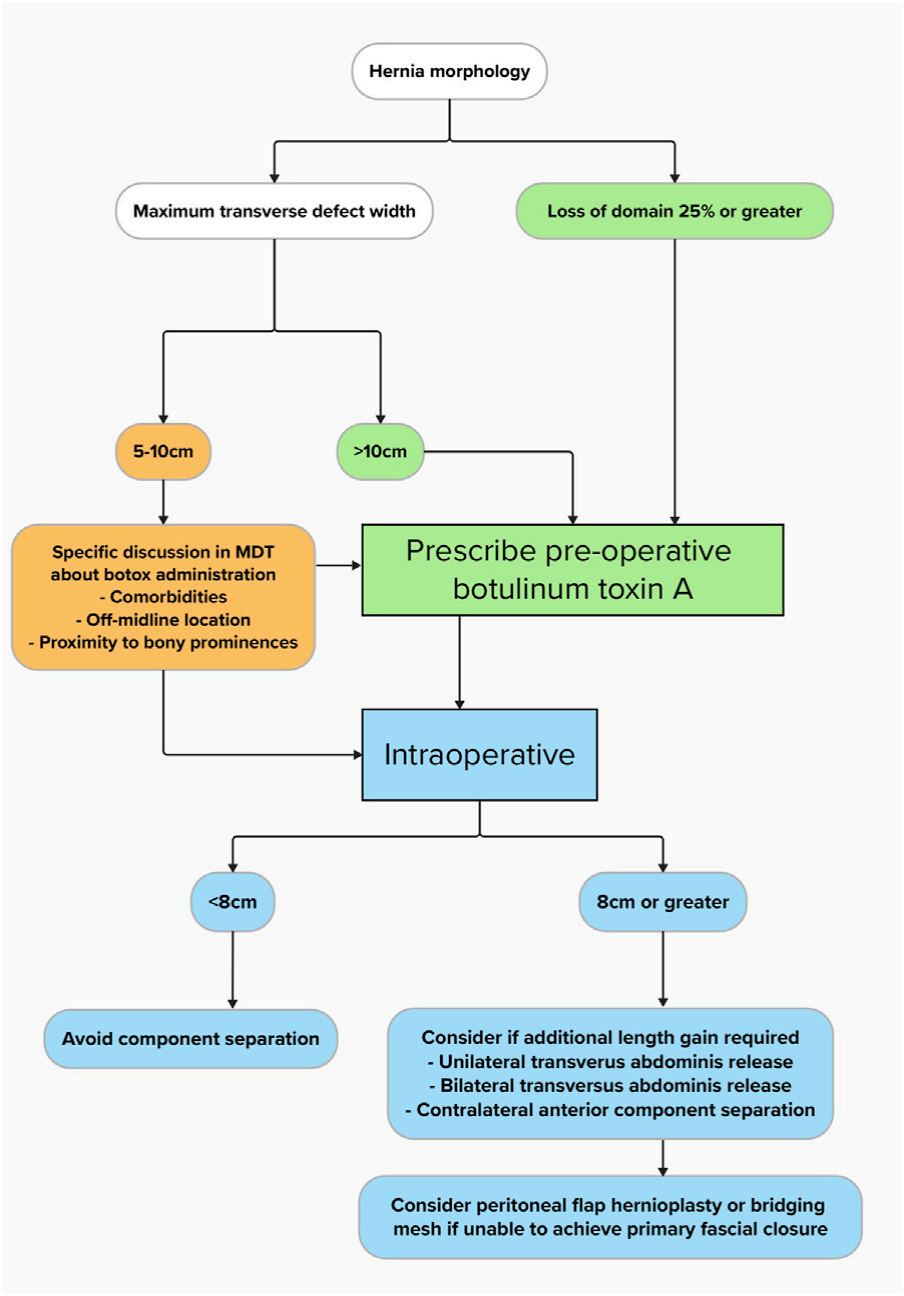
Complex ventral hernia treatment algorithm with botulinum toxin A and component separation.

Initially, the first 9 patients in this series were selected if the transverse width of their hernia defect was 10 cm or greater. However, in 2022 when our service was still in its infancy, a team from Macquarie University (Australia) published their protocol showing BTA reduced the needed for component separation. Of note, they gave BTA to patients with hernia defects down to 5 cm [6]. Based on this, our protocol was adapted to include midline defects down to 5 cm where the risk of raised intraabdominal pressure and medical comorbidities may be ameliorated by BTA administration. BTA was therefore prescribed (Botox® due to local availability) more liberally according to our updated algorithm (shown in Figure 1) and was integrated into the patient pathway and local MDT. Our MDT service is already described elsewhere [11], and consists of abdominal wall surgeons, plastic surgeons, radiologists, anaesthetists, and other key service stakeholders. These members are responsible for prescribing BTA based on the algorithm. The primary intention of BTA administration for all patients was to induce flaccid muscle paralysis in the elective setting to aid with PFC during surgery.

#### Patient Pathway and Technique for Administering BTA

To reduce strain on other services within the trust, it was important to create a pathway which facilitated patient flow using pre-existing structures, and without the requirement of additional training or staff. Patients selected for CCR were booked to attend elective theatres on a day the AWR team were operating. During a concurrent AWR case, the anaesthetic room was used as the BTA treatment room, with a dedicated anaesthetist (RJ) performing ultrasound-guided intramuscular injections according to a standardised protocol. A weight-based dose of 1% lidocaine local anaesthetic plus/minus Entonox was used for analgesia, followed by 300 IU of Botox® diluted across 150 mL of 0.9% normal saline (2 IU/mL of BTA).

BTA was divided into 3 injections per side into the external oblique, internal oblique, and the transversus abdominus muscles. Injections were placed in the anterior axillary line or at a safe point lateral to this if a giant ventral hernia encroached on it. An imaginary line was drawn between the lower border of the costal margin and upper border of the iliac crest. The first injection was placed at a point halfway along this line, followed by 2 further injections: one halfway between the first point and edge of costal margin, and one halfway between the first point and iliac crest. After administration, patients were monitored for an hour in the recovery area for adverse effects, such as bleeding, urticaria or respiratory compromise. They were then discharged home same day when there were no concerns from medical or nursing staff.

Patients were called back for their elective AWR operation between 3-4 weeks after BTA infiltration unless there were clinical or logistical factors delaying the process. Micro-Delphi process [12] was performed routinely before knife-to-skin, ensuring the MDT operative plan was central to the dialogue and possible bail-out strategies had been discussed. Patients received an open approach and where possible, placement of mesh in the retrorectus or retromuscular plane. Our protocol states that component separation may be required if the maximal transverse defect width is 8 cm or greater, the choice of which (TAR or ACS) was discussed during MDT beforehand. If tension was apparent during PFC and the retromuscular plane not previously used, then unilateral TAR was the first line choice, followed by bilateral TAR or contralateral ACS. Anterior and posterior sheaths were closed with 2/0 PDS small bites technique [13]. In extreme cases where despite component separation PFC could not be achieved, a peritoneal flap hernioplasty or bridging mesh technique was performed.

#### Clinical Governance

Clinical governance is a central facet of our tertiary AWR service to ensure outcomes are scrutinised for efficacy and patient safety. As a newly established service with off-formulary use of BTA, it was mandatory for outcomes following BTA administration to be examined at least on a yearly basis. This was the rationale for establishing a database of patients undergoing BTA injection for an AWR indication and prompted this study to be conducted. The database was divided into the following sections: baseline demographics, hernia characteristics, operative characteristics, and patient outcomes.

### Data Extraction and Outcomes

Approval from the Trust clinical governance department was granted for the retrospective collection and analysis of data for this study. Ethical approval was not required as this was conducted for service evaluation, with data captured as part of normal clinical care. Data were extracted from the Trust clinical documentation system, PowerChart, CernerWorks, and inputted onto a retrospectively maintained database stored on a secure Trust computer. All forms of documentation stored on the patient record were reviewed such as perioperative notes, clinic letters, operation notes, prescriptions, and multidisciplinary team (MDT) outcomes. Baseline demographics, preoperative MDT plan, BTA administration, surgical procedure and postoperative outcomes were of particular interest when the database was created. We defined “defect size” based on the maximum transverse width of a single defect documented radiologically or intraoperatively, or the summed total of multiple Swiss cheese defects. “Downstaging” was defined as no TAR/ACS when the defect size was 8 cm or greater. We defined “upstaging” as the opposite situation, when TAR/ACS was required with a defect <8 cm. Recurrence was detected clinically during outpatient follow-up and/or radiologically if CT scan was performed for other reasons.

### Statistical Analysis

Analysis was performed using preset summary functions on Microsoft excel (Microsoft Excel for Mac, Version 16.66.1, Microsoft Corporation, Washington, USA). Dichotomous variables were reported as number and percentage occurrence rate and continuous variables with skewed distributions were reported as median with range.

## Results

See Table 1 for a summary of patient characteristics and Table 2 for outcomes following BTA administration and AWR.

**TABLE 1:** Summary of patient characteristics.

n = 45	Value (median with range or n with %)
Demographics	
Age (years)	58 (30–80)
BMI (kg/m^2^)	32 (21–40)
Female sex (male)	22 (13)
Active smokers	7 (20%)
Diabetes	6 (17%)
ASA grade	
ASA 1	4 (11%)
ASA 2	26 (74%)
ASA 3	5 (14%)
Hernia characteristics	
Hernia defect width (cm)	8 (5–15.5)
Hernia type	
Off midline	5 (14%)
L1	2 (6%)
L3	3 (9%)
Midline	30 (86%)
M1	1 (3%)
M2	2 (6%)
M3	22 (63%)
M4	1 (3%)
M5	4 (11%)
Operative characteristics	
Component separation	18 (51%)
Anterior	5 (14%)
Posterior	13 (37%)
Unilateral TAR	9 (26%)
Bilateral TAR	4 (11%)
None (rives-stoppa only)	17 (49%)
Operating time (mins)	237 (76–338)
Mesh type	
Synthetic	19 (54%)
Biologic	16 (46%)

BMI, body mass index; ASA, American Society of Anaesthesiologists; TAR, transversus abdominis release.

**TABLE 2:** Summary of outcomes following botulinum toxin A (BTA) administration and abdominal wall reconstruction (AWR).

n = 45	Value (median with range or n with %)
General outcomes	
Length of follow up	335 (15–1047)
Time from BTA to surgery	29 (12–78)
BTA day case rate	35 (100%)
BTA complications	
Injection site complication	0 (0%)
Respiratory	0 (0%)
Cardiac	0 (0%)
Urticaria	0 (0%)
Anaphylaxis	0 (0%)
AWR complications	
SSO	11 (31%)
SSI	3 (9%)
Seroma	5 (14%)
Haematoma	1 (2%)
Superficial wound dehiscence	2 (6%)
Enterocutaneous fistula	0 (0%)
Other	
Hospital acquired pneumonia	3 (9%)
Urinary tract infection	1 (3%)
Ileus	1 (3%)
Venous thromboembolism	1 (3%)
Recurrence	1 (3%)
Re-intervention	6 (17%)
Bedside wound debridement	2 (6%)
Drainage of seroma	3 (9%) [2 by interventional radiology and 1 operative drainage]
Debridement of umbilicus	1 (2%)
Mortality	0 (0%)
Length of hospital stay	7 (5-48)

SSO, surgical site occurrence; SSI, surgical site infection.

### Baseline Demographics

During the 3-year period, a total of 45 out of the 200 patients discussed at the local MDT underwent BTA. Of these 45 patients, 35 were included in the analysis. The median age was 58 years (range 30–80), and median body mass index was 32 kg/m^2^ (21–40). Sixty three percent of patients were female (n = 22). Within the cohort, 20% were active smokers and 17% were diabetic. The majority of patients were ASA grade 2 or 3 (89%).

### Hernia Characteristics

Hernia defect widths ranged from 5cm to 15.5 cm with a median width of 8 cm. Midline hernias accounted for 86% and off-midline hernias 14%. According to the EHS classification for ventral hernia [14], the commonest location of the defect “centre-point” was M3 (n = 22), followed by M5 (n = 4) and L3 (n = 3). There were 2 hernias each for M2 and L1, and 1 hernia each for M1 and M4. Incisional or recurrent incisional hernias accounted for 91% (n = 32) of the cohort and primary hernias 9% (n = 3).

### Operative Characteristics

Overall, component separation was performed in 51% (n = 18) of cases of which 72% (n = 13) were posterior component separation and 28% (n = 5) were anterior component separation. Of those patients who had posterior component separation, 69% (n = 9) were unilateral TAR and 31% (n = 4) were bilateral TAR. Successful PFC of both anterior and posterior rectus sheaths was achieved in 91% (n = 32). A synthetic mesh (Prolene, Ethicon) was placed in 54% (n = 19) and biologic mesh (Ovitex ^®^, TELA Bio) in 46% (n = 16). Median operating time was 237 min (range 76–338).

### Outcomes

Median time from BTA administration to surgery was 29 days (range 12–78) and median length of follow-up after AWR was 335 days (range 15–1047). There were no reported complications following BTA administration, and all patients were managed as day cases. There were no re-admissions following BTA treatment.

At the point of surgery, 39% of cases (n = 9) were downstaged i.e. 39% of patients with hernia widths 8 cm or greater did not require component separation. A total of 49% (n = 17) did not require any component separation, and primary fascial closure of both anterior and posterior sheaths was achieved in 91% (n = 32) of cases. Upstaging occurred in 4 cases where the hernia width was <8 cm.

The rate of surgical site occurrence (SSO) was 31% (n = 11) and the rate of hernia recurrence was 3% (n = 1). The hernia recurrence occurred in an incisional hernia with defect width of 15 cm. Two patients required formal return to theatre–one for operative drainage of a large superficial seroma following reversal of transverse loop colostomy and bilateral TAR, and another for debridement of necrotic umbilicus after a combined AWR and abdominoplasty. The other re-interventions included bedside debridement of wound dehiscence and drainage of seroma by interventional radiology.

## Discussion

A standardised BTA protocol is a valuable tool not only in tertiary AWR units, but also in lower-volume, lower-resource settings where downstaging of complex hernias can be pivotal for patient outcomes. Whilst we acknowledge that other UK centres are using preoperative BTA, we are not yet aware of any that have published their protocols or reported on their outcomes. We found no clinically important adverse events following BTA administration in our series which aligns with existing literature [8]. Publications citing serious complications following BTA seem to be infrequent [15] and this is comparable with our experience. We therefore believe this practice is unlikely to be detrimental in the short-term given that patients typically undergo surgery within 2–3 months of administration. Whilst we failed to measure any quality-of-life outcomes such as bloating, back pain and difficulty with defaecation, these could potentially be mitigated with careful preoperative counselling. As part of our protocol, we advocate the need for centres with existing or new BTA services to audit their outcomes at least annually and review these outcomes in a local or regional clinical governance setting.

A key finding from our study is that BTA facilitated “downstaging” of the final operation in just over a third of cases which would have received component separation otherwise. This is important given that TAR is associated with increased operative time [16], higher complication rates [17], and prolonged recovery [18]. Furthermore, obviating the need for myofascial release maintains virgin planes in case of hernia recurrence and need for further surgery. While muscle elongation appears to be an objective, repeatable and quantifiable endpoint suitable for clinical trials, the concept of “downstaging” appears to be a pragmatic measure. It captures the essence of real-world intraoperative decision-making, where preoperative imaging may not fully represent the dynamic behaviour of tissues encountered during surgery. Complete PFC of both anterior and posterior sheaths together was successful in all cases except 3; two of these were midline hernias that despite component separation, required bridging of the posterior sheath with sac or omentum to restore the visceral protection layer, and one required bridging of the anterior sheath with mesh. Other studies describe a similar rate of PFC when utilising BTA protocols with or without preoperative progressive pneumoperitoneum (PPP) [6, 10, 19].

Jacombs et al. describe a protocol implemented over 7 years and routinely use BTA with hernia defects of 5 cm or greater. They found that BTA could facilitate PFC without the need for component separation when the defect was <12 cm. Our protocol was influenced by this and whilst initially using 10 cm as a cut-off point for which BTA is used, this was decreased to 5 cm if after discussion in the MDT was felt to provide benefit. These decisions were either to relieve the effects of comorbidities or pre-empt a technical challenge during surgery. Examples from our series included a patient with moderate COPD and several defects totalling 5cm, and a patient with previous renal transplant secondary to diabetic nephropathy and a 6 cm defect. In such patients, mitigating the effect of raised intraabdominal pressure was important to prevent medical complications of respiratory failure or acute kidney injury. Operative challenges included a patient with a 6 cm M1 hernia and concurrent diaphragmatic hernia following colonic interposition repair of tracheo-oesophageal fistula and a patient with multiple previous caesarean sections with a 5 cm M5 Pfannenstiel hernia. In both cases we anticipated some difficulty with PFC due tight myofascial structures close to costal margin and pelvic brim.

Our group use the Rives-Stoppa repair as our standard approach assuming the retrorectus plane has not been violated. For hernia defects of 8 cm and above, this allows component separation to be applied for additional length gain. Our data suggests that BTA may reduce the likelihood of performing component separation, which is similar to results from other studies [7, 20]. However, it is worth noting that BTA may not entirely obviate the need for component separation since some cases with defect sizes <8 cm were unexpectedly “upstaged.” These cases were all off-midline hernias or close to bony prominences where myofascial tension is greatest. The choice for which method of component separation to use is decided during the MDT but depends on multiple factors such as location of previous mesh, availability of mesh planes, rectus muscle bulk and lateral strap muscle retraction. Our preferred method of component separation is the transversus abdominis release. But this depends on good quality rectus muscle and an intact retrorectus plane. In this series, the majority of cases were incisional or recurrent incisional hernias, thus the choice of component separation could not always follow a strict algorithm but was tailored to individual circumstances.

An advantage of BTA administration is its simplicity and non-invasiveness, with ultrasound-guided injections relatively easy to learn and perform in the outpatient setting. In our unit, a dedicated anaesthetist performed BTA injections under ultrasound guidance, but this could be easily adopted by surgeons, radiologists or pain specialists depending on local expertise and resource availability. Electromyography (EMG) recording is reported in the literature [4, 7], but we believe this is impractical for routine clinical use. Ultrasonography skills are already well-established among anaesthetists who frequently perform transversus abdominis plane (TAP) blocks for other indications, whereas interpreting EMG signals is not a common skill for most medical professionals. Additionally, performing BTA injections in the anaesthetic room during an operative case presents logistical advantages. Since anaesthetists often have a trainee present who can oversee the patient undergoing surgery, BTA administration can be carried out simultaneously without disrupting workflow or need for extra procedure rooms.

In the absence of obvious contraindications such as allergy or severe neuromuscular compromise, we believe the risk benefit ratio appears to favour using BTA liberally. If an extensive dissection with increased risk of surgical site occurrences can be avoided, then it is probably worth doing. This is especially so as our team are moving towards complete extra-abdominal AWR with central zone skin excision which, anecdotally, is easier to perform due to increased soft tissue laxity. During the study period, other adjuncts to AWR such as PPP and Fasciotens® were not available and so are not described in our protocol. However, our PPP service has since developed and is used for patients with loss of domain >25% using the Sabbagh method [21]. In the future, we aim to integrate our BTA service into a perioperative care pathway which includes prehabilitation, supervised weight loss programme and progressive preoperative pneumoperitoneum (PPP) as required.

### Limitations of the Study

This study has inherent limitations. As a retrospective case series from a single institution with a relatively small sample size, its findings may not be generalisable. Although patients were prospectively enrolled, there was no randomisation and there is potential for confounding by uncategorised variables. Furthermore, downstaging and PFC depends on multiple factors, not just abdominal wall compliance, which is the primary aim of BTA. For example, PFC is dramatically affected by defect size, loss of domain, and tissue quality which we were unable to control for.

A dual-centre cohort study by Zamkowski et al. [20] was able to report on clinically significant differences in PFC rates between a BTA group and non-BTA control group of 50 patients each. In this study, patients undergoing preoperative BTA showed a significant reduction in component separation technique compared to the non-BTA group (46% and 84% respectively). Unfortunately, a notable weakness is that our study did not have a comparator arm. As the cohort grows, we plan to include a control group and perform propensity-matched scoring to allow more accurate comparison between BTA versus no BTA and reduce the influence of confounding variables. It would also be beneficial going forward to perform cost analysis in order to evaluate the economic impact of different BTA preparations and their feasibility in clinical practice.

In this cohort, we were not able to measure quality-of-life themes for patients after BTA including impact on psychology, social dynamics and daily life [22]. Several quality-of-life assessment tools have been shown to aid in a more holistic approach to patient outcomes [23] and we aim to incorporate these into future series. We are reassured however that there were no clinically important adverse events which may pose a future contraindication to BTA for eligible patients.

### Conclusion

We have established a chemical component relaxation service where BTA is now used for AWR patients with hernia defects >5 cm. In summary, our protocol for BTA administration could be effective for downstaging hernias >8 cm where component separation would otherwise be required. High quality randomised controlled trials are needed to confirm the efficacy of BTA for CCR and would benefit from strict inclusion criteria to determine the situations where it is most valuable.

## Data Availability

The original contributions presented in the study are included in the article/supplementary material, further inquiries can be directed to the corresponding author.

## References

[1] Diener MK Voss S Jensen K Büchler MW Seiler CM . Elective Midline Laparotomy Closure: The INLINE Systematic Review and Meta-Analysis. Ann Surg (2010) 251(5):843–56. 10.1097/SLA.0b013e3181d973e4 20395846

[2] Ramirez OM Ruas E Dellon AL . Components Separation Method for Closure of Abdominal-Wall Defects: An Anatomic and Clinical Study. Plast Reconstr Surg (1990) 86(3):519–26. 10.1097/00006534-199009000-00023 2143588

[3] Novitsky YW Elliott HL Orenstein SB Rosen MJ . Transversus Abdominis Muscle Release: A Novel Approach to Posterior Component Separation During Complex Abdominal Wall Reconstruction. Am J Surg (2012) 204(5):709–16. 10.1016/j.amjsurg.2012.02.008 22607741

[4] Ibarra-Hurtado TR Nuño-Guzmán CM Echeagaray-Herrera JE Robles-Vélez E de Jesús González-Jaime J . Use of Botulinum Toxin Type a Before Abdominal Wall Hernia Reconstruction. World J Surg (2009) 33(12):2553–6. 10.1007/s00268-009-0203-3 19771472

[5] Whitehead-Clarke T Windsor A . The Use of Botulinum Toxin in Complex Hernia Surgery: Achieving a Sense of Closure. Front Surg (2021) 8:753889. 10.3389/fsurg.2021.753889 34660688 PMC8517326

[6] Jacombs A Elstner K Rodriguez-Acevedo O Read JW Ho-Shon K Wehrhahn M Seven Years of Preoperative BTA Abdominal Wall Preparation and the Macquarie System for Surgical Management of Complex Ventral Hernia. Hernia (2022) 26(1):109–21. 10.1007/s10029-021-02428-2 34184138

[7] Bueno-Lledó J Martinez-Hoed J Torregrosa-Gallud A Menéndez-Jiménez M Pous-Serrano S . Botulinum Toxin to Avoid Component Separation in Midline Large Hernias. Surgery (2020) 168(3):543–9. 10.1016/j.surg.2020.04.050 32576404

[8] Timmer AS Claessen JJM Atema JJ Rutten MVH Hompes R Boermeester MA . A Systematic Review and Meta-Analysis of Technical Aspects and Clinical Outcomes of Botulinum Toxin Prior to Abdominal Wall Reconstruction. Hernia (2021) 25(6):1413–25. 10.1007/s10029-021-02499-1 34546475 PMC8613151

[9] Parker SG Halligan S Liang MK Muysoms FE Adrales GL Boutall A Definitions for Loss of Domain: An International Delphi Consensus of Expert Surgeons. World J Surg (2020) 44(4):1070–8. 10.1007/s00268-019-05317-z 31848677

[10] Yurtkap Y van Rooijen MMJ Roels S Bosmans JML Uyttebroek O Lange JF Implementing Preoperative Botulinum Toxin A and Progressive Pneumoperitoneum Through the Use of an Algorithm in Giant Ventral Hernia Repair. Hernia (2021) 25(2):389–98. 10.1007/s10029-020-02226-2 32495050

[11] Parker SG Blake H Zhao S van Dellen J Mohamed S Albadry W An Established Abdominal Wall Multidisciplinary Team Improves Patient Care and Aids Surgical Decision Making With Complex Ventral Hernia Patients. Ann R Coll Surg Engl (2024) 106(1):29–35. 10.1308/rcsann.2022.0167 36927113 PMC10757872

[12] Parker SG Joyner J Thomas R Van Dellen J Mohamed S Jakkalasaibaba R A Ventral Hernia Management Pathway; A “Getting It Right First Time” Approach to Complex Abdominal Wall Reconstruction. Am Surg (2024) 90(6):1714–26. 10.1177/00031348241241650 38584505

[13] Deerenberg EB Harlaar JJ Steyerberg EW Lont HE van Doorn HC Heisterkamp J Small Bites Versus Large Bites for Closure of Abdominal Midline Incisions (STITCH): A Double-Blind, Multicentre, Randomised Controlled Trial. The Lancet (2015) 386(Issue 10000):1254–60. 10.1016/S0140-6736(15)60459-7 26188742

[14] Muysoms FE Miserez M Berrevoet F Campanelli G Champault GG Chelala E Classification of Primary and Incisional Abdominal Wall Hernias. Hernia (2009) 13(4):407–14. 10.1007/s10029-009-0518-x 19495920 PMC2719726

[15] Zwaans WAR Timmer AS Boermeester MA . Preoperative Botulinum Toxin-A Injections Prior to Abdominal Wall Reconstruction can Lead to Cardiopulmonary Complications. J Abdom Wall Surg (2024) 3:13433. 10.3389/jaws.2024.13433 39439821 PMC11493658

[16] Riediger H Köckerling F . Limitations of Transversus Abdominis Release (TAR)-Additional Bridging of the Posterior Layer and/or Anterior Fascia Is the Preferred Solution in Our Clinical Routine if Primary Closure Is Not Possible. J Abdom Wall Surg (2024) 3:12780. 10.3389/jaws.2024.12780 38952417 PMC11215005

[17] Vasavada BB Patel H . Outcomes of Open Transverse Abdominis Release for Ventral Hernias: A Systematic Review, Meta-Analysis and Meta-Regression of Factors Affecting Them. Hernia (2023) 27(2):235–44. 10.1007/s10029-022-02657-z 35922698

[18] Zolin SJ Krpata DM Petro CC Prabhu AS Rosenblatt S Rosen S Long-Term Clinical and Patient-Reported Outcomes After Transversus Abdominis Release With Permanent Synthetic Mesh: A Single Center Analysis of 1203 Patients. Ann Surg (2023) 277(4):e900–e906. 10.1097/SLA.0000000000005443 35793810

[19] Deerenberg EB Elhage SA Shao JM Lopez R Raible RJ Kercher KW The Effects of Preoperative Botulinum Toxin A Injection on Abdominal Wall Reconstruction. J Surg Res (2021) 260:251–8. 10.1016/j.jss.2020.10.028 33360691

[20] Zamkowski M Lerchuk O Porytsky A Ushnevych Z Khomyak V Śmietański M . The Impact of Botulinum Toxin A Application on Reducing the Necessity for “Component Separation Techniques” in Giant Incisional Hernias: A Dual-Center, Polish-Ukrainian, Retrospective Cohort Study. Pol Przegl Chir (2024) 96(6):12–9. 10.5604/01.3001.0054.4919 39635752

[21] Sabbagh C Dumont F Robert B Badaoui R Verhaeghe P Regimbeau JM . Peritoneal Volume Is Predictive of Tension-Free Fascia Closure of Large Incisional Hernias With Loss of Domain: A Prospective Study. Hernia (2011) 15(5):559–65. 10.1007/s10029-011-0832-y 21584816

[22] Smith OA Mierzwinski MF Chitsabesan P Chintapatla S . Health-Related Quality of Life in Abdominal Wall Hernia: Let's Ask Patients What Matters to Them? Hernia (2022) 26(3):795–808. 10.1007/s10029-022-02599-6 35412193 PMC9003180

[23] Grove TN Muirhead LJ Parker SG Brogden DRL Mills SC Kontovounisios C Measuring Quality of Life in Patients With Abdominal Wall Hernias: A Systematic Review of Available Tools. Hernia (2021) 25(2):491–500. 10.1007/s10029-020-02210-w 32415651 PMC8055629

